# Supplementary material for: Associations between smoking to relieve stress, motivation to stop and quit attempts across the social spectrum: A population survey in England
Source: PLoS One. 2022 May 17;17(5):e0268447. doi: 10.1371/journal.pone.0268447 (PMC9113576; doi:10.1371/journal.pone.0268447)
Supplement: S1 Table — (DOCX) [file pone.0268447.s001.docx]

**S1 Table 1. Unadjusted and adjusted odds ratios for the associations of smoking to relieve stress with i) high motivation to stop and ii) quit attempts in in the next 12 months in the planned sensitivity analysis with housing tenure.**

|  | **High motivation to stop (N = 1,135)** | | | **Future quit attempt (N = 153)** | | |
| --- | --- | --- | --- | --- | --- | --- |
|  | OR | 95% CI | *p*-value | OR | 95% CI | *p*-value |
| **Smoking to relieve stress (ref = No)** |  |  |  |  |  |  |
| Yes | 1.75 | 1.26, 2.43 | **<0.001** | 1.64 | 0.86, 3.16 | 0.13 |
|  | **High motivation to stop (N = 1,135)** | | | **Future quit attempt (N = 153)** | | |
|  | OR_adj_ | 95% CI | p-value | OR_adj_ | 95% CI | *p*-value |
| **Smoking to relieve stress (ref = No)** |  |  |  |  |  |  |
| Yes | 1.48 | 1.03, 2.12 | **0.033** | 1.50 | 0.70, 3.23 | 0.3 |
| **Sex (ref = Women)** |  |  |  |  |  |  |
| Men | 0.98 | 0.68, 1.41 | >0.9 | 1.35 | 0.63, 2.94 | 0.4 |
| **Age (ref = 16-24 years)** |  |  |  |  |  |  |
| 25-34 years | 1.00 | 0.58, 1.75 | >0.9 | 0.66 | 0.13, 3.21 | 0.6 |
| 35-44 years | 0.96 | 0.53, 1.75 | 0.9 | 0.42 | 0.09, 1.91 | 0.3 |
| 45-54 years | 0.96 | 0.52, 1.75 | 0.9 | 0.51 | 0.11, 2.22 | 0.4 |
| 55-64 years | 0.89 | 0.44, 1.76 | 0.7 | 0.22 | 0.04, 1.04 | 0.06 |
| 65+ years | 0.52 | 0.24, 1.08 | 0.089 | 0.35 | 0.08, 1.56 | 0.2 |
| **SEP* (ref = Higher)** |  |  |  |  |  |  |
| Lower | 1.07 | 0.72, 1.59 | 0.7 | 1.23 | 0.54, 2.80 | 0.6 |
| **Children in the household (ref = No)** |  |  |  |  |  |  |
| Yes | 1.12 | 0.74, 1.70 | 0.6 | 0.91 | 0.36, 2.27 | 0.8 |
| **CPD**** | 0.96 | 0.93, 0.98 | **0.002** | 1.00 | 0.96, 1.05 | 0.9 |
| **Number of quit attempts in the past year (ref = 0)** |  |  |  |  |  |  |
| 1 | 5.31 | 3.52, 8.02 | **<0.001** | 5.91 | 2.14, 18.4 | **0.001** |
| 2 | 5.88 | 3.29, 10.3 | **<0.001** | 5.89 | 1.24, 42.9 | **0.040** |
| 3 | 5.10 | 2.24, 11.1 | **<0.001** | - | - | - |
| 4+ | 7.06 | 2.80, 16.9 | **<0.001** | 3.14 | 0.11, 88.0 | 0.4 |

* SEP = socioeconomic position, operationalised as housing tenure

** CPD = cigarettes per day

^ OR = Odds Ratio

OR_adj_ = adjusted for sex, age, SEP (as indicated by housing tenure), children in the household, CPD and number of quit attempts in the past year

- no individual in the follow-up sample reported 3 past-year quit attempts
