# Supplementary material for: Associations between smoking to relieve stress, motivation to stop and quit attempts across the social spectrum: A population survey in England
Source: PLoS One. 2022 May 17;17(5):e0268447. doi: 10.1371/journal.pone.0268447 (PMC9113576; doi:10.1371/journal.pone.0268447)
Supplement: S2 Table — (DOCX) [file pone.0268447.s002.docx]

**S1 Table 2.** **Unadjusted and adjusted odds ratios for the associations of smoking to relieve stress with i) high motivation to stop and ii) quit attempts in the next 12 months in the unplanned sensitivity analysis with cigarettes per day coded as a categorical variable.**

|  | **High motivation to stop (N = 1,135)** | | | **Future quit attempt (N = 153)** | | |
| --- | --- | --- | --- | --- | --- | --- |
|  | OR | 95% CI | *p*-value | OR | 95% CI | *p*-value |
| **Smoking to relieve stress (ref = No)** |  |  |  |  |  |  |
| Yes | 1.75 | 1.26, 2.43 | **<0.001** | 1.64 | 0.86, 3.16 | 0.13 |
|  | **High motivation to stop (N = 1,135)** | | | **Future quit attempt (N = 153)** | | |
|  | OR_adj_ | 95% CI | p-value | OR_adj_ | 95% CI | *p*-value |
| **Smoking to relieve stress (ref = No)** |  |  |  |  |  |  |
| Yes | 1.48 | 1.03, 2.12 | **0.035** | 1.59 | 0.74, 3.46 | 0.2 |
| **Sex (ref = Women)** |  |  |  |  |  |  |
| Men | 0.94 | 0.65, 1.35 | 0.7 | 1.39 | 0.65, 3.04 | 0.4 |
| **Age (ref = 16-24 years)** |  |  |  |  |  |  |
| 25-34 years | 1.05 | 0.60, 1.83 | 0.9 | 0.65 | 0.13, 3.22 | 0.6 |
| 35-44 years | 0.94 | 0.52, 1.72 | 0.9 | 0.43 | 0.09, 1.95 | 0.3 |
| 45-54 years | 0.97 | 0.53, 1.76 | >0.9 | 0.60 | 0.13, 2.58 | 0.5 |
| 55-64 years | 0.91 | 0.45, 1.80 | 0.8 | 0.23 | 0.05, 1.03 | 0.059 |
| 65+ years | 0.52 | 0.24, 1.09 | 0.093 | 0.36 | 0.08, 1.57 | 0.2 |
| **SEP* (ref = Higher)** |  |  |  |  |  |  |
| Lower | 0.69 | 0.48, 0.99 | **0.046** | 1.34 | 0.64, 2.81 | 0.4 |
| **Children in the household (ref = No)** |  |  |  |  |  |  |
| Yes | 1.18 | 0.78, 1.77 | 0.4 | 0.89 | 0.35, 2.20 | 0.8 |
| **CPD** (ref = 0)** |  |  |  |  |  |  |
| 1 | 0.67 | 0.44, 1.01 | 0.061 | 0.88 | 0.40, 1.92 | 0.8 |
| 2 | 0.29 | 0.05, 1.04 | 0.11 | 0.91 | 0.12, 4.68 | >0.9 |
| 3 | 1.25 | 0.18, 5.37 | 0.8 | - | - | - |
| **Number of quit attempts in the past year (ref = 0)** |  |  |  |  |  |  |
| 1 | 5.30 | 3.51, 8.00 | **<0.001** | 5.86 | 2.12, 18.3 | **0.001** |
| 2 | 6.13 | 3.43, 10.8 | **<0.001** | 5.47 | 1.15, 39.7 | **0.048** |
| 3 | 5.48 | 2.40, 11.9 | **<0.001** | - | - | - |
| 4+ | 7.16 | 2.84, 17.2 | **<0.001** | 3.40 | 0.12, 96.4 | 0.4 |

* SEP = socioeconomic position

** CPD = cigarettes per day

^ OR = Odds Ratio

OR_adj_ = adjusted for sex, age, SEP (as indicated by housing tenure), children in the household, CPD and number of quit attempts in the past year

- could not be estimated due to low cell count (i.e., no individual in the follow-up sample reported 3 past-year quit attempts and only one individual was categorised into 3 CPD)
